# Formulation of a Simulated Wastewater Influent Composition for Use in the Research of Technologies for Managing Wastewaters Generated during Manned Long-Term Space Exploration and Other Similar Situations—Literature-Based Composition Development

**DOI:** 10.3390/biotech12010008

**Published:** 2023-01-10

**Authors:** Bimi Shrestha, Rafael Hernandez, Dhan Lord B. Fortela, Wayne Sharp, Andrei Chistoserdov, Daniel Gang, Emmanuel Revellame, William E. Holmes, Mark E. Zappi

**Affiliations:** 1 Department of Chemical Engineering, University of Louisiana, Lafayette, LA 70504, USA; 2 Energy Institute of Louisiana, University of Louisiana, Lafayette, LA 70504, USA; 3 Department of Civil Engineering, University of Louisiana, Lafayette, LA 70504, USA; 4 Department of Biology, University of Louisiana, Lafayette, LA 70504, USA; 5 Department of Engineering Technology, University of Louisiana, Lafayette, LA 70504, USA

**Keywords:** space exploration, wastewater management, astronaut life support, synthetic influent formulation

## Abstract

The prospect of humans inhabiting planetary bodies is gaining interest among research and development communities, with the moon being considered as a transitory base camp and Mars the next planet humans will inhabit. NASA’s Mission to Mars program is set to have humans inhabiting Mars within on-planet space camps by the Year 2030, which has tremendously increased research and development for space exploration—including research oriented toward human life support in long-term planetary lodging camps. The sustenance of human life on Mars will not be trivial due to the unavailability of an appropriate atmosphere and usable water. This situation requires a self-sustaining human life support system that can provide the basic needs such are breathable air, potable water, food, and energy. The feasibility of sending a payload with resources adequate to support long-term human inhabitation is not reasonable, which means every resource within a Mars space camp is valuable, including human-produced wastes. A biorefinery system that treats wastewater and can also produce valuable products such as oxygen, food, and energy offers a form of circular utilization of valuable resources. To conduct research for such systems requires a wastewater influent that is representative of the wastewater to be generated by the space crew within this isolated, confined environment, which is different from what is generated on Earth due to limited variability in diet, human activity, and lifestyle in this confined area. Collection of actual wastewater influent from an isolated environment supporting humans is challenging. Additionally, to ensure a safe working environment in the laboratory and avoid the imposed threat of handling actual human feces, the proposed synthetic, non-human feces containing wastewater influent formulation offers an easy-to-produce and safer-to-handle option. This paper reviews several synthetic wastewater compositions that have been formulated for space exploration purposes. None of the formulations were found to be realistic nor adequate for a space-camp-type scenario. Thus, the formulation of a synthetic wastewater for simulating a wastewater influent from a human space-based camp is proposed in this paper. In addition, the physical, chemical, and biodegradation characteristics of the final formulation designed are presented to illustrate the value of the proposed influent formulation.

## 1. Introduction

Long-term space travel and eventually the housing of humans on other planets and the moon is foreseeable in the future. This goal implies a self-sufficient living. NASA aims to send humans to inhabit the moon by 2025 and Mars by the Year 2030, where the moon is envisioned to serve as a transitory base for longer space exploration/travels. Additionally, NASA is interested in establishing “camps” on the moon and Mars for the long-term sustainment of humans [1]. Both space travel and living within a camp will require a constant supply of human life support resources, including oxygen and water, coupled with the management of human-waste-derived wastewater influent (feces, urine, kitchen wastes, etc.).

The continuous provision of oxygen and freshwater from Earth is not available beyond the Earth’s atmosphere because it would require the use of a payload with supplies via expensive and lengthy supply flights. This would be too costly and not realistic. It is also not a sustainable choice for long-duration space travel and human-inhabited space camps on planets such as Mars. It is estimated to cost USD 10,000 to send one pound of water to the low Earth orbit, which would increase the cost by up to 40 times to send the same payload to Mars [2]. A waste model for crewed missions on a one-way Mars transit, based on the International Space Station (ISS) waste production, shows that 30% was human waste (feces and urine) and 24% was food and packaging by weight [3]. The waste in current human-inhabited space systems, such as the ISS, is collected and returned to Earth on a return payload forced into the Earth’s atmosphere, and is thus incinerated when entering the Earth’s atmosphere via entry-induced heating. However, these waste products consist of carbon and nutrients that have the potential to be recycled into useful products, but are instead burnt, thus losing the capacity of being transformed into life-sustaining materials and/or hydrogen-based fuel. The onsite generation of water, oxygen, and nutrients from wastewater can reduce payload mass by over 90% [4]. This focuses new research on the potential reliance on the waste generated by the crew for producing life-sustaining products via treatment and recycling. Therefore, it is evident that the wastewater will need to be efficiently treated to deem it fit for human reuse/consumption. This requires extensive work in the research and development of space travel studies inclusive of treating envisioned generated wastewater influents. The use of municipal wastewater influents that are generated on Earth would not be representative of what is generated by an isolated, confined space crew that are subjected to a uniform diet, carefully controlled hygienic products, restricted human activities, managed cooking, controlled wastage, and controlled water usage, resulting in a much different biophysicochemical character compared to a typical Earth-based wastewater influent. Therefore, it is recommended to use a synthetic wastewater influent that would be less character-fluctuating as compared to human-population-based wastewater from the municipal treatment plants on Earth. Additionally, synthetic wastewater reduces concerns over pathogen and disease exposure to technology developers within laboratory and pilot support environments.

Several technologies have been evaluated in the past by NASA and others for treating wastewaters generated on orbitory, transit, and sited camp scenarios [5,6,7]. Both abiotic and biotic systems have been evaluated and continue to be further developed. For long-term camp scenarios, often, biotic systems, such as for aerobic and anaerobic biotreatment, are proposed, but need further testing and development. These studies show that much more research is needed to provide appropriate treatment within a treatment system confined by a reduced weight payload allowance. However, more importantly, circular waste management (recycle) schemes are needed in order to use the waste constituents as feedstocks for energy and oxygen production among other potential beneficial life support uses.

There are several waste streams expected to be generated within an isolated human space camp. Grey wastewater typically consists of toilet flush water, and sink and shower water, and has been formulated in the past [8,9]. Wastewater primarily consists of human feces; however, real human feces and urine use is not encouraged by the CDC due to safety concerns when handling [10]. Additionally, exposure to actual human-based wastewater has become of greater concern due to the global COVID-19 pandemic. The use of biohazard-certified facilities and rigorous safety training, in addition to the risk posed when using actual human feces, is one of the reasons for the development of a synthetic wastewater influent that does not actually contain human-based feces and urine. A second reason is that the composition of the synthetic wastewater influent must closely imitate the wastewater and its composition expected to be generated by astronauts in the confined, isolated space camps. As discussed above, municipal wastewater influents will poorly simulate the expected composition of the influents of interest for space camp studies.

This study focused on three work stages. The first was to review the literature on synthetic wastewater influents and simulant feces for purposes including, but not limited to, space exploration to serve as the basis to formulate the composition of a low-strength wastewater influent that can be utilized for human life support within space research. This study then goes further and proposes a formulation of a synthetic wastewater influent, based on the literature reviewed and our decisional rationalization of the data, for use in studies evaluating wastewater treatment within space travel and planetary human space camp life support operations. Finally, this body of work also tested the developed wastewater influent formulation via chemical analysis, and the use of a biodegradation assay using anaerobic biotreatment as the test bioprocess. The biodegradation assay was conducted using batch-fed laboratory digester units to assess its utilization to determine biotreatment potential. Both the resulting chemical composition and the ability of the proposed wastewater influent to be biodegraded were considered a critical proving step to the acceptance of the resulting final influent formulation. Biotreatment was selected as the treatment step because it is considered to be the most likely process that will be used in a human planetary space camp.

## 2. Review of Human Feces Composition

Human feces have been simulated for research and development purposes such as long-term space travel, diaper production, hospitals, and the management of health sector wastes [10]. Animal feces, such as monkey and dog feces, and chicken litter, have been used in the past [10]; however, the physical, chemical, and microbial properties of human feces differ from animal feces. Human feces have a median pH of 6.64 and are mostly comprised of water (74.6%), bacterial biomass (25–54% dry solids) of the organic fraction, and the inorganic fraction of the feces is mostly undigested diet [11,12]. A study on the preparation of synthetic feces by Wignarajah and Litwiller shows that human feces consist of 65–85% water and 15–35% solids. The major chemical components of human feces (dry weight) are shown in Table 1, which reports that carbohydrates and fat make up to 50% of the feces [10]. Similarly, fecal simulant formulated by Kaba, et al. consisted of (in % dry weight) 33% cellulose, 25% torpulina, 7% *E. coli*, 10% casein, 20% oleic acid, 2% KCl, 2% NaCl, and 1% CaCl_2_ [13]. A new formulation based on this recipe replaced *E. coli* with yeast extract, and oleic acid with peanut oil, which consists of 40–80% oleic acid. A compilation of simulated human feces based on these studies is presented in Table 2, and these have been used to prepare synthetic wastewater influents [10,13,14,15]. In general, it can be stated that human feces are composed of mainly carbohydrates, proteins, and yeast, comprising 40–50% of the feces, and chloride salts in smaller amounts (<10% *w*/*w*).

## 3. Review of Synthetic Urine Composition

Humans produce one to four liters of urine on average per day, and thus, it is a major component in wastewater systems. Urine is waste that remains after the reabsorption of over 95% of salts and water by the body, and is mostly composed of water, salts, metabolic byproducts, and toxins [16]. A study by Volpin, et al. on urine treatment at the international space station found higher calcium in urine due to the lifestyle in a space station, leading to changes in renal adaptation induced by bone stress in microgravity and lower urine output. The main salts in urine are calcium oxalate, calcium phosphate, magnesium ammonium phosphate(struvite), uric acid, sodium acid urate, ammonium acid urate, and cystine [16]. Synthetic urine formations have been reported in the literature, and are compiled in Table 3 [8,17,18]. The majority of the urine is composed of urea, followed by chlorides and sulfates, with smaller amounts of ammonia [19]. These findings recommend that synthetic wastewater must include urea, ammonium salts, and chlorides in the form of potassium, magnesium, and calcium.

## 4. Review of Previously Proposed Synthetic Wastewater Influent Compositional Formulations

Synthetic wastewater influents have been developed based on ersatz (i.e., artificial or synthetic) recipes that have been prepared to study the treatment of ISS wastewaters. The chemical composition of the ersatz wastewater influent has been prepared from the data received from an integrated water recovery system test at the Johnson Space Center for an early planetary base (EPB) wastewater stream [9]. A recipe for ersatz wastewater has been formulated by Veerostko and Carrier that has the characteristics of wastewater generated on a transit mission and an EPB [9]. The ersatz water chemistry and physical properties, such as pH, total organic carbon (TOC), total inorganic carbon (TIC), chlorides, phosphate, and ammonia nitrogen, are provided in Table 4. The wastewater includes organic and inorganic urine concentrates diluted 10 times, humidity condensate (captured from perspiration and breathing air that is 10 times diluted), and Sabatier condensate from air regeneration [8]. The transit wastewater is more concentrated and has lower pH in comparison to the EPB wastewater due to the lower production of wastewater per person per day during transit. The production of wastewater during astronaut transit is expected to be 3.53 L, whereas the EPB, being a stationary camp scenario, is expected to produce 11.85 L of wastewater per person per day, which is due to the inclusion of shower, handwash, humidity condensate, and Sabatier product resulting in a higher dilution factor, hence higher pH (Table 1A, Veerostko and Carrier [9]). This ersatz recipe has been modified to dilute the COD:N ratio by Chen, et al. for a membrane reactor, and the composition is listed in Table 5 [20]. The recipe for synthetic municipal wastewater has been reviewed, the results of which are reported in Table 6 [21,22,23,24]. The carbon, nitrogen, and phosphorus ratios in the formulation vary according to research needs and the feedstock used. The wastewater consists of organic carbon sources with the majority being in the form of glucose, starch, and cellulose. The nitrogen and ammonia sources in the wastewater are provided by urea, ammonium chloride, and peptone. Phosphates and carbonates provide phosphorus and inorganic carbon in the wastewater. The presence of salts in the urine proposes the presence of potassium, calcium, and magnesium salts in the composition of synthetic wastewater.

## 5. Design of a Synthetic Wastewater Influent Formulation

After reviewing the literature on simulated human feces, urine, and synthetic wastewater influents that is inclusive of space research and municipal wastewater, a synthetic wastewater was designed and formulated, as presented in Table 7. Since some studies may aim to vary the strength of the wastewater influent, the proposed formulation was designed to serve as a stock/base composition that can be diluted as needed. Based on our literature review and knowledge of wastewater influents, this formation needs to be diluted 20 times to be considered indicative of our expectations of a wastewater influent to be generated within a Mars human space camp, which is expected to be of low strength, as measured using the chemical oxygen demand (COD) test (soluble COD < 500 mg/L and total COD < 1000 mg/L). This stock solution strategy will allow the researcher to target a specific strength by diluting to any targeted COD level, which allows for the testing of a treatment system over a range of conditions. The formulation is composed of starch, glucose, sodium acetate, and peanut oil (42% wt%) as the main carbon source derived from simulated urine, feces, and synthetic wastewater. The wastewater formulation also consists of cafeteria food waste that, in majority, is comprised of a green salad and cabbage blend, which serves as a source of cellulose. Yeast extract is also included to substitute for the *E. coli*. urea, which is the major component in urine, has been included and is a source of nitrogen and organic carbon in the wastewater. For macronutrients, Triple 20 (N-P-K) fertilizer is used for providing nitrogen, phosphorus, and potassium. Ammonium chloride and peptone have also been included as a source of nitrogen and protein, respectively. Minerals such as potassium, calcium, and magnesium, which are represented in urine, have also been included in amounts less than 5% by weight. Milk powder in the form of casein provides additional protein in the synthetic wastewater formulation. Dog food and dog feces have been included in the composition to represent human feces since actual human feces were decided not to be included in the formulation (see the introductory section of this paper). It has been reported that the microbial composition in mammals is related to the food intake. Dogs have been human companions for centuries, thus they have been feed similar food constituents (a review of a typical dog food composition substantiates this assumption), which suggests that dog digestion likely has a close resemblance to human digestion [25]. Similarities in the microbiome using taxonomic profiling have been reported between dogs and humans [25]. The dog feces were collected from a single dog, fed commercial dog food. Dog food contributes high protein, carbohydrates, and minerals. Bile secreted by the human liver and present in human feces has been added to the formulation in the form of ox bile nutritional supplement pills.

## 6. Characteristics of Synthetic Wastewater Based on Analytical Testing

The synthetic wastewater formulation was prepared in filtered water, and the parameters of interest are pH, chemical oxygen demand (COD), total carbon (TC), total inorganic carbon (TIC), total organic carbon (TOC), total nitrogen (TN), total ammonia nitrogen (TAN), and reactive phosphorus (orthophosphates). The resulting concentrations of these key characteristics/component levels are reported in Table 8 based on our in-house analyses of the resulting influent formulation. The pH of the human feces, urine, and food waste averaged in the range of 6.2–6.8. The soluble COD is the COD of filtrate after passing the sample through a 0.45-micron filter, and is thus lower than the total COD, which contains particulates and hence has a higher COD value. The total organic carbon is measured as the difference between the total carbon and total inorganic carbon, and is lower than the COD.

## 7. Biodegradation Assay Testing of the Proposed Influent

**Figure 1:**
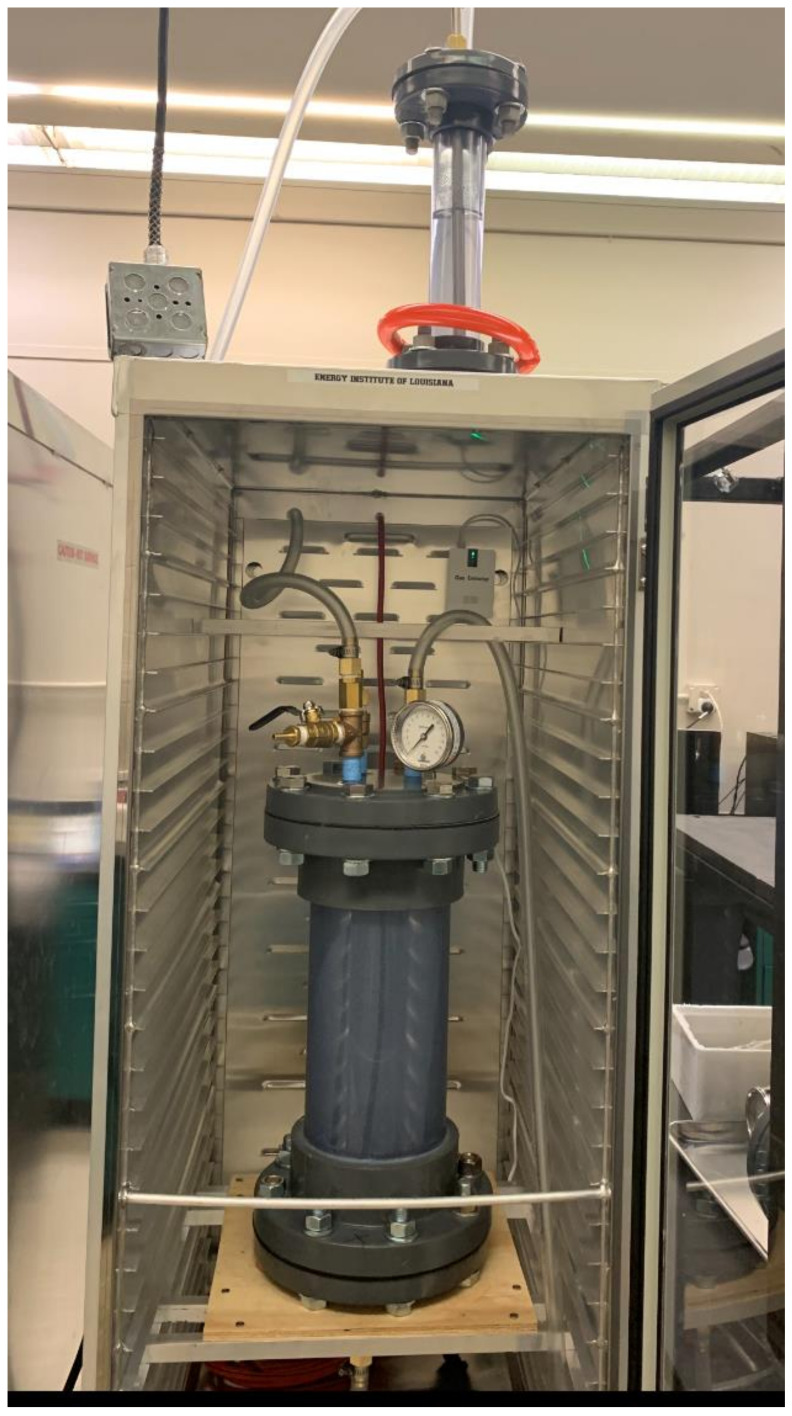
Laboratory-scale (8 L) digester unit for anaerobic digestion of synthetic wastewater.

**Figure 2:**
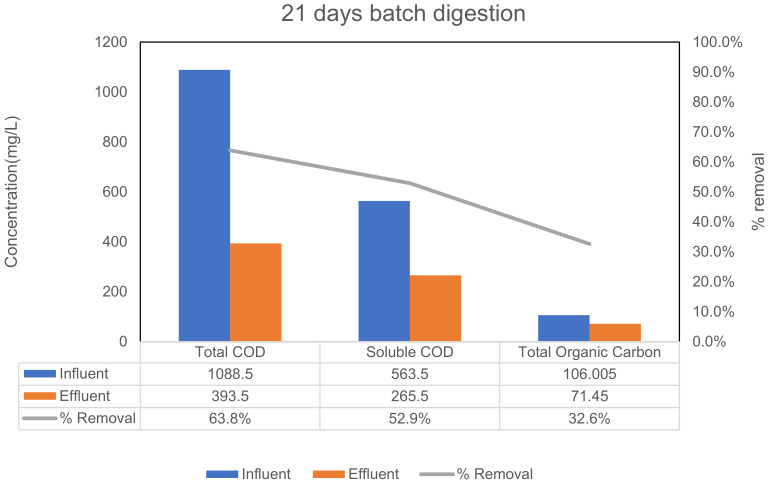
Degradation of chemical oxygen demand and total organic carbon using proposed synthetic wastewater using anaerobic batch digestion.

In order to evaluate the viability of the proposed synthetic wastewater formulation as a potential biotic treatment influent, biotreatment assay testing was performed using anaerobic digestion as the biotreatment process. Thus, a duplicate set of bioassays to test the biodegradation potential of the formulated wastewater influent was performed using a batch operated anaerobic digester system. Our laboratory routinely performs these assays as part of our ongoing anaerobic digestion R&D. The synthetic wastewater influent for these biotreatment tests was prepared using the developed stock formulation from Table 7 with a 20X dilution, and was seeded with anaerobic sludge obtained from an active anaerobic digester located within a local municipal wastewater treatment facility. Anaerobic digestion at mesophilic temperature of 37 °C was applied using duplicate 8 L batch-fed digesters (6.5 L working volume), as shown in Figure 1. The anaerobic microbial seed was added to the digesters at 3% (v/v, with an initial 1% solids concentration as collected) with the reactor volume being the synthetic wastewater influent. The chemical oxygen demand of the wastewater influents were measured using Hach TNT COD kits, which follow the standard method for analysis of wastewater according to APHA. The total organic carbon of the samples was measured using a Shimadzu TOC analyzer. The levels of total chemical oxygen demand (tCOD), soluble chemical oxygen demand (sCOD), and total organic carbon (TOC) removed after 21 days of batch digestion are provided in Figure 2. It is to be noted that the influent level in the experiment refers to SWW, including the 3% seed, which increases the initial concentration of the desired parameters compared to the SWW only. The reduction in COD and TOC levels proves the biodegradability of the synthetic wastewater using anaerobic digestion. The low-strength wastewater, after being anaerobically digested, produces valuable products such as biogas and organic acids (which serve as platform chemicals), and the removal of organic pollutant deems this formulation appropriate for applicability in space research and technologies. The sCOD and TOC removal through batch and fed-batch digestion are calculated using the following formulae:

(1) $$\mathrm{COD}\;\mathrm{removal}\;\%\;=\frac{\mathrm{Influent}\;\mathrm{COD}-\mathrm{Effluent}\;\mathrm{COD}}{\mathrm{Influent}\;\mathrm{COD}\;}*100\%\;$$

(2) $$\mathrm{TOC}\;\mathrm{removal}\;\%=\frac{\mathrm{Influent}\;\mathrm{TOC}-\mathrm{Effluent}\;\mathrm{TOC}}{\mathrm{Influent}\;\mathrm{TOC}\;}*100\%$$

The batch-fed digestion of the synthetic wastewater shows the removal of soluble COD to be 53%, which is within the accepted range based on our experience with these assay types. Over 30% of the total organic carbon was removed, which was also expected for the digestion system used (21-day incubation). Various operational strategies, pretreatment methods, and their combinations could be applied to increase the removal of the organic pollutants from this synthetic wastewater if desired, but that was not the focus of this present work. As expected, the influent performed as planned by showing good removal characteristics, and thus, provides a reasonable simulant for the purposes of wastewater treatment process performance testing on space-based influents.

## 8. Conclusions

A synthetic wastewater influent formulation dedicated for research on the treatment/management research of long-term space missions and/or human space camps is designed and presented in this paper. The wastewater generated by space crews during lunar and Mars missions is anticipated to be composed of mainly human feces, urine, and food waste/residue, which will differ in characteristics from the wastewater generated on Earth. The use of wastewater produced on Earth cannot be used for space research since the variations in the diet, lifestyle, and activities have a direct effect on digestion and human excreta; therefore, the generation of a wastewater possessing different characteristics and sources is proposed. In addition to the variability of wastewater attributes, the lack of resources, mainly water, and the need for a waste treatment system for space colonization by humans, treatment of wastewater while producing potable water is crucial. The results from both the analytical tests the anaerobic digestion assay of the presented formulation provide strong evidence of the biodegradability of synthetic wastewater and the presence of expected compositional chemicals, thus making it a good influent for space-based wastewater treatment tests.

## Figures and Tables

**Table 1:** Major components of human feces based on chemical composition.

Major Components of Human Feces Based on the Chemical Composition (Dry Weight)	
Fat content	5–25%
Carbohydrate (fiber)	10–30%
Nitrogenous material	<2%
Minerals	5–8%
Bacterial debris	10–30%
Protein content	10–15%

**Table 2:** Composition of simulated feces based on several studies.

	(i)	(ii)	(iii)	(iv)	(v)
	%W	%W	% W	g/L	gram
Cellulose	6	10	15	380	600
Polyethyleneglycol	3		15		
Peanut oil	11		20	200	
Miso	17	17.5	15		
Potassium Chloride	2	2	10	40	40
Calcium Chloride	1	1			30
Water	60			1500	
Monocalcium Phosphate				10	
Yeast		30		380	
Torpulina					430
*E. coli*			30		120
Casein					170
Oleic Acid		20			370
Sodium Chloride		2			40
Ground, Dried Vegetable				50 mg	
Inorganics			5		
Psyllium		17.5			

(i) Wang et al. [14], (ii) Penn et al. [15], (iii) and (iv) Wignarajah & Litwiller [10], and (v) Kaba et al. [13].

**Table 3:** Synthetic urine composition.

Synthetic Urine Composition					
	Colon, et al. (g/L) [17]	Urine 1 (g/L) from Hintze, et al. [8]	Urine 2 (g/L) from Hintze, et al. [8]	Urine 3 from Hintze (g/L) [8]	Sarigul, et al. (g/L) [18]
Urea (CH_4_N_2_O)	14.2	52.021		5.2	15.00
Creatinine (C_4_H_7_N_3_O)	3.00	5.221		0.52	0.881
Ammonium citrate (C_6_H_11_NO_7_)	2.00	12.34		1.23	
Sodium Chloride (NaCl)	8.00		23.126	2.31	1.756
Potassium Chloride (KCl)	1.65		5.436	0.54	2.308
Potassium bisulfate (KHSO_4_)	0.50				
Magnesium Sulfate (MgSO_4_)	0.20				
Potassium dihydrogen phosphate (KH_2_PO_4_)	1.75		1.069	0.11	
Potassium bicarbonate (KHCO_3_)	0.50		2.197	0.22	
Histidine, soluble (C_6_H_9_N_3_O_2_)		0.958		0.1	
Taurine (C_2_H_7_NO_3_S)		0.556		0.06	
Glutamic acid (C_5_H_9_NO_4_)		1.66		0.17	
Uric Acid (C_5_H_4_N_4_O_3_)					0.250
Glucose (96%) (C_6_H_12_O_6_)		2.636			
Ammonium formate (97%) (NH_4_HCO_2_)		1.466		0.15	
Ammonium oxalate monohydrate (C_2_H_10_N_2_O_5_)		0.665		0.07	
Trisodium citrate dihydrate (Na_3_C_6_H_5_O_7_.2H_2_O)					0.720
Magnesium chloride hexahydrate (MgCl_2._6(H_2_O))			5.483	0.55	
Potassium carbonate (K_2_CO_3_)			0.474	0.05	
Potassium sulfate (K_2_SO_4_)			7.424	0.74	
Calcium Chloride (CaCl_2_)			0.221	0.02	0.185
Sodium sulfate (Na_2_SO_4_)			4.144	0.41	1.700
Ammonium Chloride (NH_4_Cl)					1.266
Potassium oxalate monohydrate (K_2_C_2_O_4_.H_2_O)					0.0350
Magnesium sulfate heptahydrate (MgSO_4_.7H_2_O)					1.082
Sodium phosphate monobasic dihydrate (NaH_2_PO_4_.2H_2_O)					2.9212
Sodium phosphate dibasic dihydrate (Na_2_HPO_4_.2H_2_O)					0.831

**Table 4:** Water chemistry on transit wastewater and early planetary base ersatz [9].

Ersatz	Transit Wastewater	Early Planetary Base Wastewater
pH	2.6 ± 0.2	8.9 ±0.2
TOC (mg/L)	2209 ± 221	631 ± 63
TIC (mg/L)	0	391 ± 59
Chloride (mg/L)	1870 ± 281	514 ± 77
Phosphate (mg/L)	75 ± 11	116 ± 17
Ammonium-N (mg/L)	221 ± 33	852 ± 128

**Table 5:** Components of the modified NASA early planetary base wastewater ersatz [20].

Component	Quantity (Per L)	Component	Quantity (Per L)
Shampoo (suave for kids)	275 mg	Acetic acid	34 mL
Ammonium bicarbonate	2.3 g	Benzoic acid	1 mg
Ammonium hydroxide	300 mg	Benzyl alcohol	5 µL
Ammonium citrate	370 mg	Ethanol	6.5 mL
Ammonium formate	45 mg	Acetone	0.5 µL
Ammonium oxalate monohydrate	20 mg	Caprolactam	4 mg
Sodium chloride	690 mg	Phenol	0.6 mg
Potassium chloride	200 mg	N, N-dimethylformamide	0.7 µL
Sodium bicarbonate	200 mg	Ethylene glycol	3.3 µL
Potassium phosphate monobasic	166 mg	4-Ethyl morpholine	1.5 µL
Potassium sulfate	690 mg	Formaldehyde	3 µL
Urea	160 mg	Formic acid	4.4 µL
Lactic acid	80 mg	Methanol	7.6 µL
Creatinine	160 mg	1,2-Propanediol	2 µL
Histidine	29 mg	2-Propanol	2.4 µL
Taurine	17 mg	Propionic acid	15 µL
Glutamic acid	51 mg		
Glucose	78 mg		

**Table 6:** Review of synthetic municipal wastewater.

Synthetic Municipal Wastewater Composition (mg/L)				
Component	I	II	III	IV
Starch	20,000	122		
Sodium Acetate	20,000	131.6		
Urea	15,000	91.74		
Peptone (Pancreatic)	6000	17.4		
Powdered Milk	3000	116		
Soy Oil	3000	29		
Fertilizer (L)	200			
Magnesium Hydrogen Phosphate Trihydrate		29		
Potassium Phosphate Dibasic				260
Potassium Phosphate Monobasic		3.14		520
Ferrous Sulfate Heptahydrate		5.8		
Yeast		52.24	100–300	
Manganese Sulfate Monohydrate		0.11		20
Ammonium Chloride		12.75	170	
Glucose				
Monosodium Phosphate			37	
Magnesium Sulfate				100
Calcium Chloride				20
Magnesium Chloride Monohydrate			9	
Potassium Chloride			25	
Sucrose				400
Ammonium Sulfate				200
Sodium Carbonate				400
Ferric Chloride hexahydrate				20
Trace metals		A	1 mL of B	

A. (mg/L) Cr(NO_3_)_3_.9H_2_O 0.77, CuCl_2_.2H_2_O 0.536, MnSO_4_.H_2_O 0.108, NiSO_4_.6H_2_O 0.336, PbCl_2_ 0.1, ZnCl_2_ 0.208. B. (mg/L) H_3_BO_3_ 50, FeCl_2_.4H_2_O 2000, ZnCl_2_ 50, MnCl_2_.4H_2_O 500, CuCl_2_.2H_2_O 30, (NH_4_)6MoO_7_.4H_2_O 90, NiCl_2_.6H_2_O 50, Na_2_SeO_3_. 5H_2_O 100, EDTA 1000, Resazurin 500,36% HCl 1 mL. References: I Mavioso & Galvao [21], II Nopens et al. [22], III Wiegant & Lettinga [23], IV Irvine & Yang [24].

**Table 7:** Synthetic wastewater formulation (stock solution).

Component	g/L
Cafeteria Food Waste	3
Peanut Oil	2
Urea	1
Starch	3
Glucose	2
Yeast Extract	0.075
Dried Dog Food	0.150
Dog Feces	2
Peptone	0.05
NPK Fertilizer	1
Sodium Acetate	0.15
Ammonium Chloride	0.020
Sodium Chloride	0.030
Potassium Chloride	0.040
Calcium Chloride	0.030
Ferrous Sulfate Heptahydrate	0.002
Magnesium Hydrogen Phosphate Trihydrate	0.012
Potassium Phosphate Monobasic	0.001
Casein (Milk Powder)	0.005
Bile (ox)	0.010

**Table 8:** Characteristics of the synthetic wastewater (diluted 20 times).

Feed	mg/L
pH	6.6
Soluble COD	345 ± 75
Total Carbon (TC)	150 ± 15
Total Inorganic Carbon (TIC)	22 ± 7
Total Organic Carbon (TOC)	133 ± 20
Total Ammonia Nitrogen (TAN)	15 ± 8
Reactive Phosphorus	17 ± 4
Total Nitrogen (TN)	34 ± 3

## Data Availability

Data are available.
